# Early Indication of Decompensated Heart Failure in Patients on Home-Telemonitoring: A Comparison of Prediction Algorithms Based on Daily Weight and Noninvasive Transthoracic Bio-impedance

**DOI:** 10.2196/medinform.4842

**Published:** 2016-02-18

**Authors:** Illapha Cuba Gyllensten, Alberto G Bonomi, Kevin M Goode, Harald Reiter, Joerg Habetha, Oliver Amft, John GF Cleland

**Affiliations:** ^1^ Personal Health Solutions Philips Research Eindhoven Netherlands; ^2^ Department of Electrical Engineering Eindhoven University of Technology Eindhoven Netherlands; ^3^ Department of Health Professional Studies Faculty of Health & Social Care University of Hull Kingston-Upon-Hull United Kingdom; ^4^ ACTLab University of Passau Passau Germany; ^5^ National Heart & Lung Institute Imperial College London United Kingdom

**Keywords:** Heart failure, telemonitoring, deterioration detection, alert algorithms, ambulatory monitoring, impedance

## Abstract

**Background:**

Heart Failure (HF) is a common reason for hospitalization. Admissions might be prevented by early detection of and intervention for decompensation. Conventionally, changes in weight, a possible measure of fluid accumulation, have been used to detect deterioration. Transthoracic impedance may be a more sensitive and accurate measure of fluid accumulation.

**Objective:**

In this study, we review previously proposed predictive algorithms using body weight and noninvasive transthoracic bio-impedance (NITTI) to predict HF decompensations.

**Methods:**

We monitored 91 patients with chronic HF for an average of 10 months using a weight scale and a wearable bio-impedance vest. Three algorithms were tested using either simple rule-of-thumb differences (RoT), moving averages (MACD), or cumulative sums (CUSUM).

**Results:**

Algorithms using NITTI in the 2 weeks preceding decompensation predicted events (*P*<.001); however, using weight alone did not. Cross-validation showed that NITTI improved sensitivity of all algorithms tested and that trend algorithms provided the best performance for either measurement (Weight-MACD: 33%, NITTI-CUSUM: 60%) in contrast to the simpler rules-of-thumb (Weight-RoT: 20%, NITTI-RoT: 33%) as proposed in HF guidelines.

**Conclusions:**

NITTI measurements decrease before decompensations, and combined with trend algorithms, improve the detection of HF decompensation over current guideline rules; however, many alerts are not associated with clinically overt decompensation.

## Introduction

Chronic heart failure (HF) is common [1] and a substantial drain on scarce healthcare resources [2]. Much of the costs of HF are due to the high rate of unplanned admissions for worsening HF. For patients who survive an admission for worsening HF, rehospitalization rates are high and >20% will die within one year [3,4]. Furthermore, the high prevalence and costs associated with HF are projected to rise as the population ages [5]. Telemonitoring could reduce costs and improve outcomes [6] by substituting infrequent assessments at a clinical facility by a health professional with frequent remote monitoring done by patients themselves. This could facilitate more timely and tailored interventions. The efficacy of telemonitoring would be greatly improved if decompensation events could be detected before the onset of severe symptoms [7,8].

Worsening heart failure may lead to weight gain as a consequence of fluid retention and edema and, if uncorrected, can lead to hospitalization and ultimately death. The Heart Failure Association of America (HFSA) and the European Society of Cardiology (ESC) guidelines both recommend daily weight monitoring. The ESC recommends that patients experiencing a weight increase of 2 kg or more in 3 days should alert healthcare professionals and increase their diuretic dose [9]. The HFSA recommends the restriction of sodium and water after an increase of more than 2 lbs (0.9 kg) in 1 day, or more than 4 lbs (1.8 kg) over a week, followed by an alert to healthcare professionals if the increase continues [10].

Worsening hemodynamics with increased vascular resistance, afterload mismatch, congestion, and diastolic dysfunction are thought to precede fluid accumulation [11]. Increased end-diastolic pulmonary arterial pressure (PAP), a direct measure of hemodynamic overload, and decreased intrathoracic impedance (ITI), an indirect measure of pulmonary congestion, have both been observed in the days and weeks prior to decompensation [12-14]. Thoracic impedance can also be measured noninvasively (NITTI) [15], which correlates with ITI [16], making measurement possible in a far broader range of patients. NITTI measures a much larger field; however, the variability in measurements may depend on the patients’ willingness and ability to position electrodes accurately. Recently, several new wearable devices have been proposed for this purpose, such as specialized vests [17,18] or adhesive patches [19,20].

An increased risk of decompensation has been shown for both weight gain [21] and decline in ITI [22]; however, recent studies have shown that absolute changes in weight over short time periods are not sensitive in detecting impending decompensation [23-25], and that ITI may have high sensitivity but a high rate of false alarms per patient-year [26]. However, to the authors’ knowledge, recently proposed prediction algorithms comparing body weight and impedance head-to-head have not been investigated using *noninvasive* technology.

The aim of this investigation was to evaluate and compare the predictive value of previously published algorithms using measurements of daily body weight, and noninvasive measures of NITTI from a smart-textile vest, to detect decompensation prior to the onset of severe symptoms leading to hospitalization.

## Methods

### Patient Population

The data for this analysis were collected as part of the MyHeart heart failure management observational study [27]. The MyHeart study was unique in its collection of several different vital signs and innovative markers using noninvasive sensors and a home-telemonitoring system. Six HF clinics in Germany and Spain participated in the collection of the clinical data. Patients were included in the study if they had chronic HF with an elevated N-terminal of the prohormone brain natriuretic peptide (NT-proBNP ≥ 500 pg/ml), were taking at least 40 mg/day of furosemide or an equivalent, and were in the New York Heart Association (NYHA) functional class II, III, or IV. They were excluded if they had the following: severe chronic obstructive pulmonary disease (COPD GOLD Class > 2), primary pulmonary hypertension, renal insufficiency requiring dialysis, a psychiatric or neurological disorder of moderate to severe degree (eg, dementia, schizophrenia, substance disorder, psychotic depression), prior acute myocardial infarction or coronary artery bypass grafting (CABG) in the previous 3 months. Ethical approval was provided by the Medical Ethics Committees in the 2 respective countries.

Of 148 patients recruited from October 2008 to July 2010, 108 had the system installed and data recorded; 3 did not fit the criteria, 3 were unavailable at installation, 1 died before installation, and 33 withdrew before system installation. Of the remaining 108 users, 17 used the system on less than 30 occasions, leaving 91 patients as the focus of this exploratory analysis. Their mean (SD) age was 63 (12) years and 64 were men. Mean weight was 84 (19) kg, mean BMI was 29 (6) kg/m^2^, and mean left ventricular ejection fraction (LVEF) was 31 (12) %. Most patients had mild (NYHA class II: 60%) or moderate (NYHA class III: 36%) symptoms. Etiology was ischemic in 47%, idiopathic dilated cardiomyopathy in 31%, valvular disease in 5%, and other in 9%. Comorbidities included hypertension (68%), diabetes (37%), atrial fibrillation (36%), renal dysfunction (28%) and COPD (13%). Treatment included angiotensin converting enzyme (ACE) or angiotensin receptor blockers (ARB) (87%), beta-blockers (88%), MRA (53%), diuretics (84%), digoxin (21%), and implantable cardioverter-defibrillator/cardiac resynchronization therapy (ICD/CRT) (23%/14%). The average monitoring time was 10 months, during which 19 patients were hospitalized one or more times due to decompensated HF, with a total of 24 decompensated HF hospitalizations. The adverse events were adjudicated by an advisory committee.

### Daily Measurements of Body Weight and NITTI

**Figure 1:**
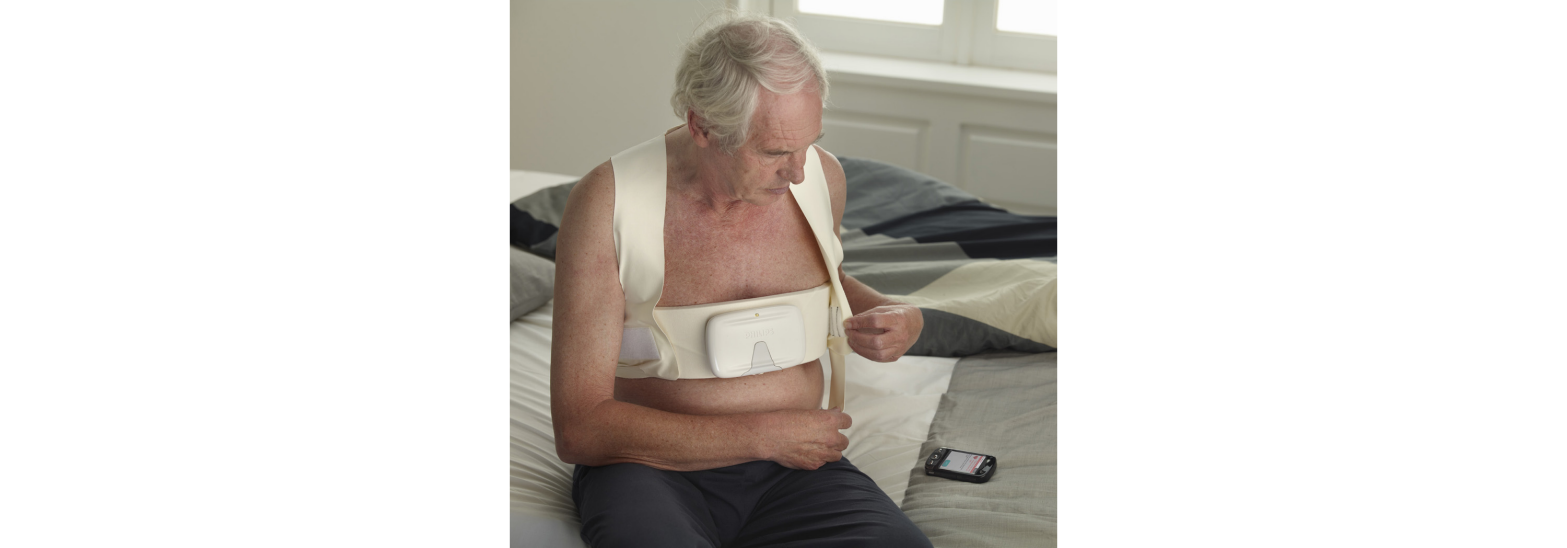
The bioimpedance vest shown by a model subject correctly applying it across the chest. Textile electrodes on each side of the flexible measurement panel inject currents at different frequencies and register the resulting voltage to calculate the impedance parameter relating to extracellular fluid volume.

Patients were instructed on how to perform measurements of body weight and NITTI. Measurements were carried out in the morning before eating breakfast. Body weight was collected using a weight scale (Philips Medical Systems, Andover, Massachusetts, USA), which automatically logged the measurements (accuracy ± 0.1 kg). TTI was measured using a wearable bio-impedance vest [28], shown in Figure 1. The vest measures TTI at several electrical frequencies (10 kHz-1MHz). These recordings give a characterization of the electrical properties of the tissue, as described by the Cole-Cole model [29]. At low measurement frequencies, biological tissue impedance is mainly determined by the extracellular fluid content and characteristics. At higher frequencies, electrical properties are determined by both the intracellular and extracellular fluid content. Multi-frequency measurements of thoracic bio-impedance therefore allow isolation of the Cole parameters that indirectly reflect either the intracellular or extracellular fluid content. We used the *external resistance* derived from the Cole-Cole model, since this indirectly reflects extracellular water, which is the component associated with decompensation. In another study, we have shown that this metric tracks changes in symptoms and fluid loss during treatment for decompensated HF [17].

### Alarm and Event Definition

The weight and NITTI data were applied to published algorithms (detailed description in Multimedia Appendix 1), to predict the onset of decompensation prior to subsequent hospitalization due to worsening heart failure. The output of these algorithms, the *output index*, could be as simple as the difference between the current measurement and the measurement made 2 days previously, or a more complex calculation (eg, one based on cumulative sums). An alert is triggered when the output index exceeds a specific threshold.

The predictive power of the algorithms was assessed by exploring their ability to alarm within a prespecified period before a hospitalization due to worsening heart failure. Changes in NITTI are thought to precede changes in weight prior to hospitalization [12,21]. Depending on the measure used, previous studies have considered alerting periods from 2 weeks [23] up to one month [30] before hospitalization. In this study, a 2-week period was chosen as an adequate period before a hospitalization, during which alarms should be raised, giving time for the patient or clinician to act. Alerts occurring outside of this period were counted as false alarms. Short periods of a few days at the start of monitoring, end of monitoring, and directly following a hospitalization did not fit into any 2-week division and were subsequently removed from the analysis.

### Performance Assessment of Algorithms

**Figure 2:**
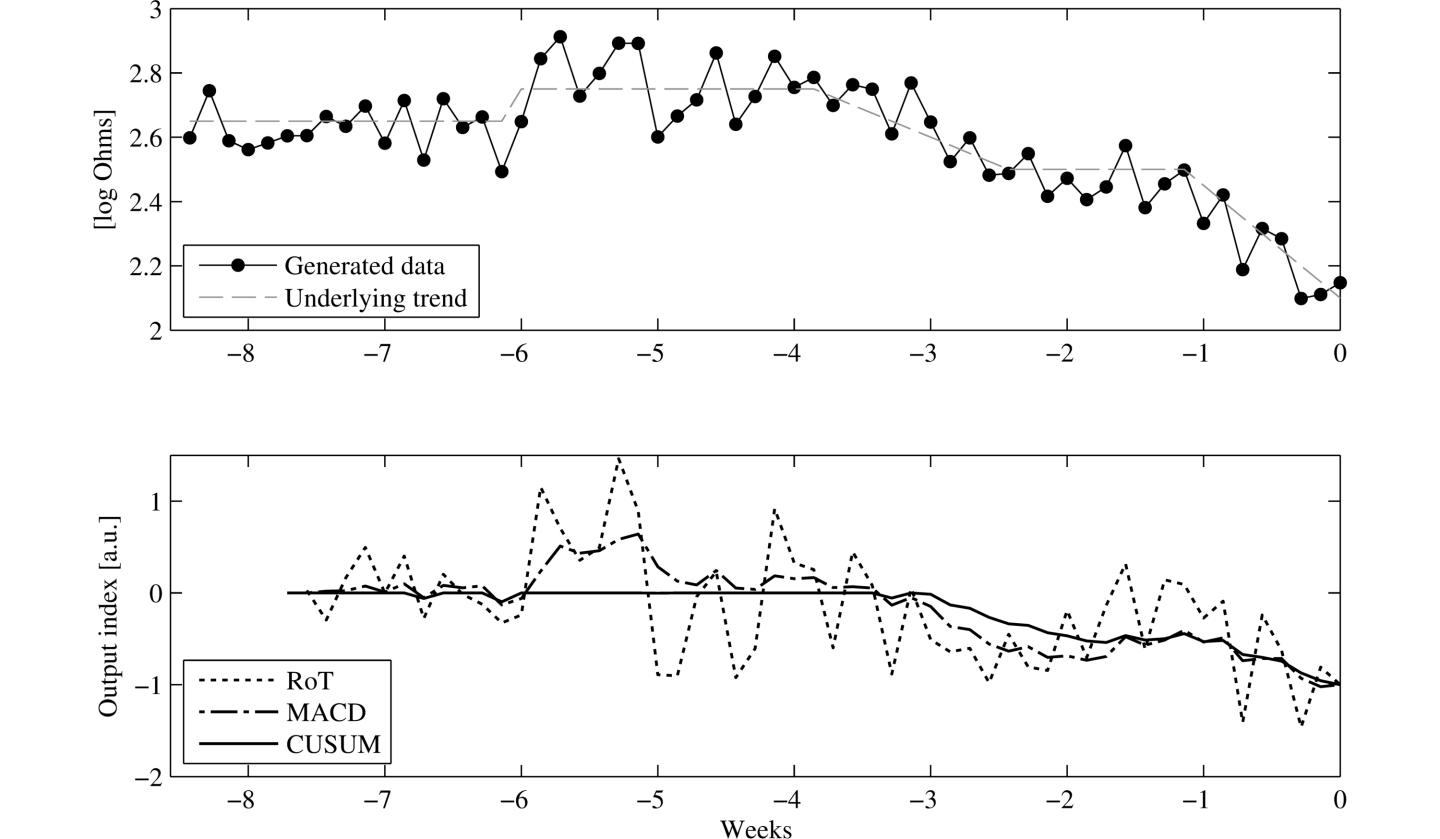
Generated example data with the underlying trend in NITTI are shown in the top graph. The resulting output of the three algorithms, normalized to the last measure to show the qualitative difference between the algorithms, is shown in the bottom graph.

Three types of alert algorithms are compared in this study: rule-of-thumb (RoT) [21,23,26], moving average convergence divergence (MACD) [23], and cumulative sum control chart (CUSUM) [31]. The qualitative differences between these are shown in Figure 2. Rule of thumb (RoT) methods provide a noisy measure for which chance readings have a large effect, sometimes with no underlying trend; however, they also provide a fast response to changes. Moving averages (MACD) react more slowly but follow underlying trends better, in both directions. Cumulative sums (CUSUM) provide uni-directional detection and lead to longer sustained alerts. For a detailed description of the definitions of each algorithm see Appendix 1. The predictive performance of the algorithms was compared using receiver operator curve (ROC) analysis. The sensitivity and specificity of each algorithm was calculated by dividing the measurement data into periods of 2 weeks, in such a way that a period containing a decompensated hospitalization would end when the hospitalization event occurred. This led to the following definitions:

1. *True positive*: An alarm during the 2 weeks preceding a hospitalization;

2. *False positive*: An alarm during any other 2-week period;

3. *True negative*: A 2-week period without any alarms;

4. *False negative*: A 2-week period ending in a hospitalization without any alarms.

### Algorithm Selection and Optimization

Each of the algorithms considered in this study (RoT, MACD, CUSUM) have modifiable parameters that will alter their behavior and ultimately their predictive performance. We tested the performance of each algorithm for a range of possible parameter values. For the RoT algorithms, the number of days (*d*) between the measurements used to calculate the difference was varied from 1 day to 21 days. In the MACD algorithm, the long-term average parameter *N*_*l*_ was varied between 10 and 50 days in increments of 5 days, and the short-term moving average parameter *N*_*s*_ was varied between 1 and 10 days. In the CUSUM algorithm, the parameter determining the length of the running mean and standard deviation (*d*) was varied between 10 and 30 days in increments of 5 days, and the parameter determining the depreciation of the accumulated sum (*c*) was evaluated between 0.5 and 1.5, in increments of 0.2.

Segmentation of the data into 2-week periods results in substantially more periods without an HF-related hospitalization compared to those with one. To avoid producing algorithms that raise a large number of false positive alarms, previous studies have focused only on alarms with high specificity [20,30]. In this investigation, the best parameters were chosen to be those that maximized the area under the curve for thresholds with a specificity >95%. The output index for each algorithm was then normalized to allow the correct estimation of the ROC curves during the cross-validation procedure described below.

Parameter optimization can lead to models that overfit the data, which then would not generalize well to other data sets. To minimize these effects, we implemented a stratified leave-patient-out cross-validation (CV) method for the parameters in the RoT, MACD, and CUSUM algorithms. This procedure randomly splits the data into 8 groups, while maintaining the number of patients and decompensation events in each group. The parameters were then optimized for the data with one group left out. The data from the left-out group were then used to evaluate the performance of the optimized parameters. This was repeated until all groups had been left out once. The left-out groups were then recombined to provide an unbiased ROC curve. The optimal threshold for the output index was chosen to be the Youden point with specificity larger than 90%.

### Statistics

Comparisons between the recorded measurements and the output index for the different algorithms in the 2 weeks preceding hospitalization and all other periods were tested with a mixed-effect model using patient specific intercepts as random effects. An arbitrary significance of 0.05 was assumed throughout. Missing data due to adherence issues were removed from the analysis by excluding periods in which less than 3 [32] measurements per week were found. In the case of algorithms that needed previous data points to estimate trends, a linear imputation between adjacent data points was carried out. It should be noted that when the algorithms processed the data, imputations were only made on data that would have been available for a system running in real time; no imputations using future values were done. NITTI measurements were log-transformed to adjust for skewness. All listed algorithms were developed and evaluated using the software suite MATLAB 7.13.0.564.

## Results

### Data Characteristics

Among the 91 patients for whom data were included in the analysis, 24 heart failure-related hospitalizations occurred in 19 patients. Of the 24 hospitalizations, 9 had less than 3 weekly weight recordings and 12 had less than 3 weekly impedance recordings preceding the hospitalization, and were excluded from the analysis. The minimum window for the CUSUM algorithm excluded an additional 2 for its analysis.

### Prediction Performance

The predictive performance of guideline-based rules and published algorithms using weight are presented in Table 1. With the exception of those rules with very low specificity (ie, <60%), all rules based on short-term increases had low sensitivity when applied to the data (typically <25%). Rules based on longer-term increases showed higher sensitivity; however, only one had a specificity >90%. The MACD algorithm with the parameter proposed by Zhang et al. [23] outperformed the other weight algorithms.

**Figure 3:**
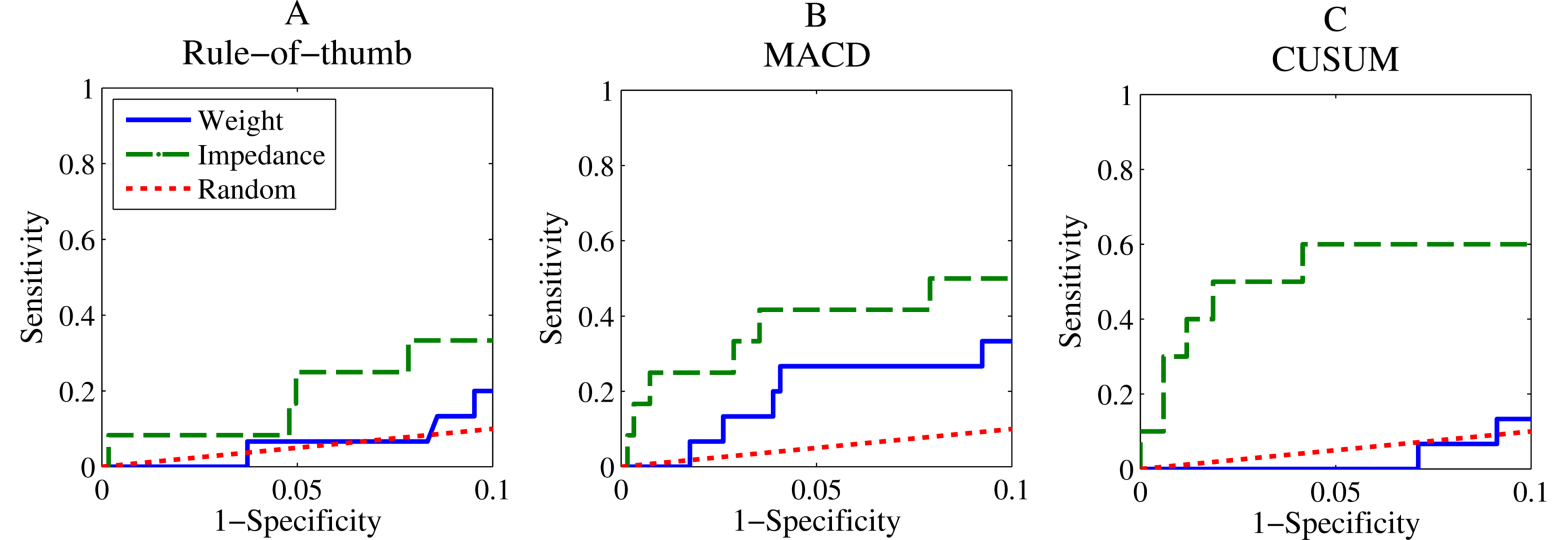
ROC curves from the cross-validated evaluation for the three considered algorithms in the specificity range from 0.9 to 1. A shows the rule of thumb algorithm, B the MACD algorithm, and C the CUSUM algorithm. Performance using NITTI measures is shown with the dashed green line, weight is shown with the blue line, and random chance is portrayed by the red dotted line.

The cross-validation analyses of the developed models based on published algorithms are presented in Figure 3. The RoT-based algorithms using weight have poor sensitivity at a specificity between 90-100%, with performance close to random chance. This poor sensitivity was also observed when evaluating previous published guidelines using windows between 2 and 3 days (Table 1). As expected, this sensitivity increased when longer windows and/or lower thresholds were used, but at the cost of a lower specificity.

The MACD algorithm improved performance for both weight and impedance. The CUSUM algorithm improved performance for NITTI. The performance of trend algorithms was superior to previously published algorithms (Table 1).

**Table 1:** Performance of different weight algorithms in anticipating an upcoming decompensation.

Source	Weight algorithm	Sensitivity %	Specificity %	PPV^a^ %	NPV^b^ %
Guideline issuing bodies	>2 lbs^c^ in 1 day [10]	67	56	1.4	99.5
	>2 kg in 3 days [9]	13	87	0.9	99.1
	>4 lbs^c^ in 1 week [10]	27	87	1.8	99.2
Existing literature	Random chance	50	50	0.9	99.1
	>2 lbs in 1 day or >3 lbs in 3 days [26]	73	50	1.3	99.5
	>2 lbs in 1 day or >5 lbs in 3 days [26]	67	56	1.4	99.4
	>3 lbs in 1 day or >5 lbs in 3 days [26]	13	82	0.7	99.1
	>3 lbs in 1 day or >7 lbs in 3 days [26]	7	83	0.4	99.0
	>4 lbs in 1 day or >7 lbs in 3 days [26]	7	93	0.9	99.1
	>4 lbs in 1 day or >9 lbs in 3 days [26]	7	93	0.9	99.1
	>5 lbs in 1 day or >9 lbs in 3 days [26]	0	100	—	99.1
	>2 lbs in 1 week [21]	80	45	1.3	99.6
	>5 lbs in 1 week [21]	20	94	2.7	99.2
	>4 lbs in a 5 to 80 days MACD^d^ [23]	20	97	6.3	99.3

^a^PPV: positive predictive value

^b^NPV: negative predictive value

^c^To convert to kilograms multiply by 0.45

^d^MACD: moving average convergence divergence

### Optimal Parameters

**Figure 4:**
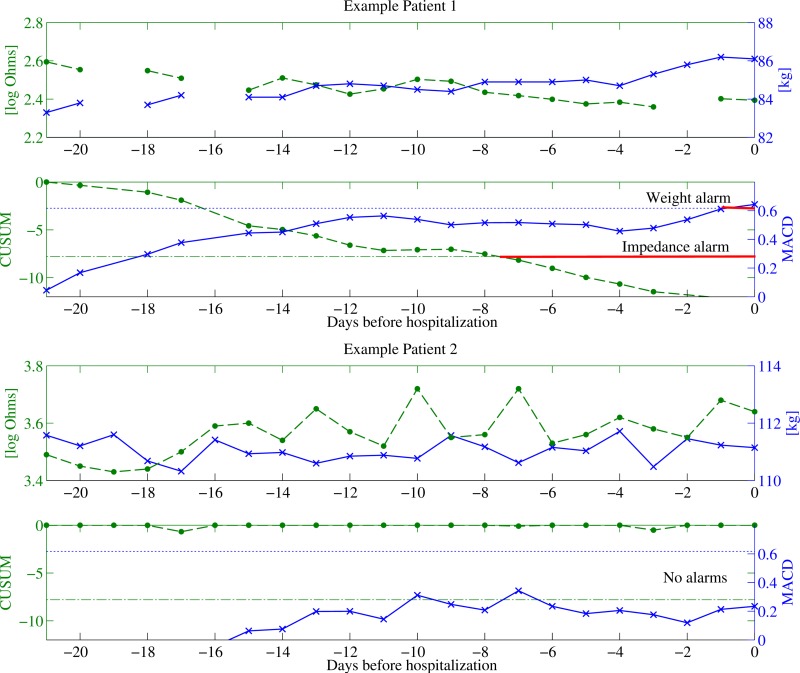
Three weeks of telemonitoring data from two patients with high compliance before an upcoming decompensation. Circles correspond to NITTI measurements and the NITTI-CUSUM algorithm and crosses correspond to weight measurements and the weight-MACD algorithm. Optimal thresholds are shown as dash-dotted lines in green for NITTI and dotted blue lines for weight.

The output of the 2 best performing algorithms for weight and impedance with optimal parameters (maximum Youden index with specificity >90%) is shown in Figure 4. Clear trends in both weight and impedance can be seen for Patient 1 and both algorithms managed to alert before the decompensation; a full week in advance for impedance and a day in advance for weight. Patient 2, on the other hand, had no or weakly visible trends, which were not enough to trigger an alert. The patient did exhibit large daily weight fluctuations, which could have indicated instability; however, this was not picked up by the algorithms. The optimal parameters for all 3 algorithms for weight and impedance are shown in Table 2, together with the cross-validated performance measures. Both trend algorithms using NITTI outperformed the weight algorithms.

**Table 2:** Cross-validated performance measures of the algorithms at the maximum Youden index within a specificity of 90-100%.

Optimal algorithms^a^		Sensitivity %	Specificity %	PPV^b^ %	NPV^c^ %
**Weight**					
	RoT^d^: >2.7 kg in 17 days	20	90	1.95	99.2
	MACD^e^: >0.62 kg (N_s_=9, N_l_= 20 days)	33	91	3.2	99.3
	CUSUM^f^: >8.7 with 10-day average, c=0.75	13	91	1.4	99.1
**NITTI** ^g^					
	RoT: <-0.27 (log ohm) in 21 days	33	92	4.2	99.2
	MACD: <-0.059 (log ohm) (N_s_=9, N_l_= 35 days)	50	92	5.9	99.5
	CUSUM: <-7.8 with 20-day average, c=0.75	60	96	10.9	99.6

^a^The optimal parameters and thresholds were estimated from the full data (for stability and variance of cross-validated parameters and thresholds, see Table 3).

^b^PPV: positive predictive value

^c^NPV: negative predictive value

^d^RoT: rule of thumb

^e^MACD: moving average convergence divergence

^f^CUSUM: cumulative sums

^g^NITTI: noninvasive transthoracic bio-impedance

### Algorithmic Stability

The use of a cross-validation procedure to minimize biased performance measures generated several plausible parameters for the tested algorithms; these are presented in Table 3. In general, RoT had lower variance in estimated parameters than MACD, which in turn had lower variance than CUSUM, coinciding with the increasing complexity of the algorithms. Parameter variance was especially high for the weight CUSUM algorithm, which could explain the poor performances when compared to MACD.

Mean values for weight, impedance, and the respective output indices of the optimal algorithms during periods preceding a hospitalization compared to the other periods are shown in Table 4. A statistically significant difference was only found for the NITTI measurements and algorithms based upon NITTI.

**Table 3:** Mean, standard deviation, and individual values for the estimated optimal parameters in each of the 8 folds created using the described stratified cross-validation procedure.

Measure		Body weight								Transthoracic impedance							
CV ^a^ step		1	2	3	4	5	6	7	8	1	2	3	4	5	6	7	8
**RoT** ^b^																	
	Threshold	3.5 (0.08)								-0.31 (0.035)							
		3.5	3.56	3.4	3.56	3.45	3.6	3.4	3.4	-0.3	-0.31	-0.3	-0.3	-0.3	-0.3	-0.3	-0.4
	Days	14.4 (3.7)								20.5 (1.41)							
		11	17	11	17	17	20	11	11	21	17	21	21	21	21	21	21
**MACD** ^c^																	
	Threshold	0.8 (0.38)								-0.10 (0.014)							
		1.59	0.62	0.31	0.62	0.62	0.97	0.62	0.95	-0.12	-0.1	-0.1	-0.1	-0.1	-0.09	-0.09	-0.13
	Short-term avg. window	8.6 (1.19)								8.1 (0.99)							
		8	9	8	9	9	10	9	9	9	8	8	8	8	9	9	6
	Long-term avg. window	25.6 (10.84)								36.3 (3.54)							
		50	20	15	20	25	30	20	25	45	35	35	35	35	35	35	35
**CUSUM** ^d^																	
	Threshold	11.0 (7.87)								-8.13 (2.65)							
		30	8.7	8.7	8.7	6.9	8.1	8.7	8.1	-7.8	-10.3	-7.8	-7.8	-11.1	-4.40	-11.14	-4.64
	Days	26.9 (18.3)								18.8 (2.31)							
		50	10	10	10	40	40	10	45	20	20	20	20	15	20	15	20
	Depreciation	1.13 (0.40)								0.75 (0.19)							
		1.5	0.75	0.75	0.75	1.5	1.5	0.75	1.5	0.75	0.75	0.75	0.75	0.50	1	0.50	1

^a^CV: Cross-validation

^b^RoT: rule of thumb

^c^MACD: moving average convergence divergence

^d^CUSUM: cumulative sums

**Table 4:** Population mean output index values for RoT, MACD, and CUSUM algorithms using the optimal parameters (see 2) in the 2-week period preceding a hospitalization compared to all other periods.

Measure	Mean (SD) value in 2-week period before decompensation	Mean (SD) value in nondecompensation periods	Statistical significance^d^
Weight (kg)	83 (10)	84 (19)	.97
Weight-RoT ^a^ (kg)	0.3 (1.2)	0.06 (0.87)	.76
Weight-MACD ^b^ (kg)	0.08 (0.30)	0.02 (0.22)	.24
Weight-CUSUM ^c^ (kg)	1.9 (2.7)	0.8 (1.3)	.58
TTI (log Ohm)	3.0 (0.3)	3.4 (0.3)	<.001
TTI-RoT (log Ohm)^a^	-0.07 (0.12)	0.00 (0.08)	<.001
TTI-MACD (log Ohm)^a^	-0.032 (0.044)	0.003 (0.028)	<.001
TTI-CUSUM (log Ohm)^a^	-6.4 (9.4)	-0.7 (2.0)	<.001

^a^RoT: rule of thumb

^b^MACD: moving average convergence divergence

^c^CUSUM: cumulative sums

^d^Estimated with a mixed-effect model with patient specific random effects. For the algorithms the cross-validation output was used.

## Discussion

### Principal Findings

The main finding of the present study is that change in NITTI is a stronger predictor of an impending decompensation compared to changes in weight (cross-validation estimate was 60% for NITTI-CUSUM vs 33% for Weight-MACD) and that both measurements benefit from trend detection algorithms. Mean values of NITTI in the 2-week period preceding a decompensation event were lower than in nondecompensation periods (*P*<.001).

Fluid overload is one of the leading causes for HF hospitalization and body weight increase has been linked to an increased risk of hospitalization [21]. However, directly applying a weight gain difference to predict imminent decompensation is challenging. This study corroborates the findings of Zhang [23] and Abraham [26], who also reported low predictive ability of alarms using short-term weight change. Short-term weight increase will detect a large and rapid fluid accumulation. Our evaluation of the rule suggested by the ESC guidelines is that it has high specificity but it is not a very sensitive method to predict HF hospitalization, as gradual weight increases are missed. A moving average algorithm focuses on progressive changes in weight, removing much of the inherent variability in weight measurements and errors due to the home setting in which patients might deviate from the measurement protocol, and daily changes due to dietary and fluid intake are averaged out. This could explain why lower threshold values led to higher sensitivity while still retaining specificity.

The increase in thoracic fluid due to congestion should decrease impedance measurements. Several studies have reported positive results from algorithms using impedance to detect decompensations [19,20,33]. To test algorithms proposed for decompensation detection using impedance measurements, we employed a cross-validation procedure to estimate performances. The results are similar, although on the lower side of what has been reported for ITI in terms of sensitivity (76.4% [26], 76.9% [33], 60% [34]), perhaps partly accounted for by the robust methods we employed. Reported performances from feasibility studies usually decline in later prospective studies [35], which the leave-subject-out protocol is designed to emulate.

Comparisons between predicted performances of weight and impedance measurements in Figure 3 show that impedance is the stronger predictor. This is also suggested by the analyses of the mean output index in the 2 weeks preceding a decompensation (Table 4), for which a statistical difference was found compared to periods without decompensation for all impedance algorithms as well as the impedance value, but not for any of the weight algorithms. Abraham et al. [26] also showed a higher sensitivity for impedance measurements when compared to weight. However, we showed that the gap in performance could be made smaller with more sophisticated weight trend algorithms compared to the rules suggested by Abraham (in which the 3 rules with a specificity >90% had a maximum sensitivity of 7%). Sensitivity to fluid build-up in the lungs, whether through redistribution of fluids or retention, could explain the increased performance of impedance when compared to body weight [11]. Similarly, weight loss from malnutrition might mask fluid accumulation in weight measurements, which would still be picked up by NITTI. The focus in this study on high specificity algorithms might also have put weight algorithms at a slight disadvantage; evidence of this can be found in the stability analysis (Table 3), in which the high parameter variance for the weight-CUSUM algorithm could have resulted from the difficulty of finding a highly specific algorithm, which led to a negative impact on its cross-validated performance.

The difficulty in assessing prediction algorithms is known [36]. Different evaluation metrics can show diverging results, because they shed light on different aspects of performance. Definitions of what constitutes a true positive and false positive have a great effect on performance. In this study, we focused on algorithms with high specificity evaluated using 2-week intervals, with the best-performing alarm having a sensitivity rate of 60%. Although this catches several patients at a high specificity, it still raises unexplained alarms and has a relatively low positive predictive value of 10.9% for impedance and 3.2% for weight. A measure focusing on the workload associated with managing these alerts, such as false alarms per patient year has been used by several other studies as a surrogate specificity metric [26,33-35]. Defined as an alert not resulting in a hospitalization, the NITTI-CUSUM algorithm has a cross-validated estimate of 0.48 false alarms per patient year. These seemingly contradictory performance measures can be explained by the rarity of 2-week periods resulting in hospitalization, when compared to the full amount of telemonitoring data. An alarm that goes on for 5 weeks would cross three 2-week periods and could generate 3 false positives; however, using the false-alarm metric it would only add one false alarm.

Therefore, the positive predictive value of 10.9% should be seen in the context of 2-week windows having both high specificity and sensitivity and compared to the relatively low predictive value of current weight algorithms.

Low levels of positive predictive value have also been observed in many other studies evaluating prediction algorithms from daily measurements [35,37,38]. The concept of predicting future events might be less realistic than providing indications that could be acted upon. This approach could tailor actions depending on which monitored sign was detected. Indeed, many signs that have been linked to deterioration, for example, arrhythmias [39], breathing rates [38], and heart-rate variability [40], can be detected noninvasively and may be included in such an approach. Importantly, the implementation of better decompensation algorithms will reduce the number of clinical alerts that would need to be dealt with by a telehealth nurse or physician. This will result in better resource utilization, with the management of larger patient caseloads and, therefore, a reduction in the costs of patient management.

### Limitations

Although clinicians were blinded to the observational data, they could have intervened based on increased weight data for worsening patients. If such interventions did not result in a hospitalization, they were not recorded in this study and might have negatively affected the results. In the SENSE-HF trial [37], a substantial increase in positive predictive value was reported after including signs and symptoms of worsening HF diagnosed by a physician rather than only adjudicated HF hospitalizations; therefore, it could also be expected that several false positives were due to “mild” decompensations. Indeed, it is possible that patients often self-correct decompensation by reducing dietary salt, increasing adherence to medication, or even by taking extra doses of diuretic. Changes in environmental temperature might also affect compensation. In this study, high specificity alerts were explored. However, sacrificing specificity for improved sensitivity may be a good complement if management of alerts can be handled by patients without resorting to professional advice. Combining specific alerts with a strategy of health maintenance might be superior to one of only crisis detection and management [41]. Most patients are interested and able to contribute to their care if they are given the information and confidence to do so. Remote monitoring provides a safe environment or safety net to encourage such behavior.

Incorrectly using the measurement equipment could have caused erroneous values with the net effect of lowered performances. The surface on which the scales sit, their accuracy, clothing, and use by other family members can all cause problems with measurement. Bio-impedance weight scales (a different technology from NITTI) require patients to remove their socks and shoes and hence may improve the consistency of measurement. Giving patients feedback and asking them to recheck their weight if it falls out of the expected range are all likely to improve the data quality on which the algorithms are based. The limited amount of data available for this study makes generalizations difficult. Application of cross-validation procedures were employed to minimize this effect; however, the calculated percentage values were ultimately derived from a small set of subjects and should therefore be seen as qualitative indicators of performance.

### Conclusion

Daily measurements of transthoracic impedance using a vest with textile electrodes is a feasible way to monitor HF and provides a more accurate indication of upcoming decompensations when compared to weight for all 3 algorithms tested (RoT, MACD, and CUSUM). Trend detection algorithms outperformed RoT measures suggesting that tracking the progression is more important than direct measures of change, which currently are suggested by guidelines.

However, the low positive predictive value of all the algorithms tested did not allow accurate prediction of impending HF hospitalizations. Implementation of trend detection algorithms might better serve as indications of worsening, which, when integrated with other clinical measures, could be useful for treatment management. The promising results from this investigation warrant further trials with noninvasive TTI as a technology for the management of HF, perhaps connected to actionable alerts. These alerts would promote a strategy of “health maintenance” to keep the patient as close to their ideal state as possible on a daily basis, which could be combined with a strategy of “crisis detection and management” if the first strategy failed.

## Appendix

Multimedia Appendix 1 Detailed description of algorithms to detect decompensated HF.

## Abbreviations

ACE: angiotensin converting enzyme
ARB: angiotensin receptor blockers
CABG: coronary artery bypass grafting
CUSUM: cumulative sums
CV: cross-validation
ESC: European Society of Cardiology
HF: Heart Failure
HFSA: Heart Failure Association of America
ICD/CRT: implantable cardioverter-defibrillator/cardiac resynchronization therapy
ITI: intrathoracic impedance
LVEF: left ventricular ejection fraction
MACD: moving average convergence divergence
NYHA: New York Heart Association
NITTI: noninvasive transthoracic bio-impedance
NPV: negative predictive value
PAP: pulmonary arterial pressure
PPV: positive predictive value
ROC: receiver operator curve
RoT: rule-of-thumb
