# Supplementary material for: Early Indication of Decompensated Heart Failure in Patients on Home-Telemonitoring: A Comparison of Prediction Algorithms Based on Daily Weight and Noninvasive Transthoracic Bio-impedance
Source: JMIR Med Inform. 2016 Feb 18;4(1):e3. doi: 10.2196/medinform.4842 (PMC4777885; doi:10.2196/medinform.4842)
Supplement: Multimedia Appendix 1 [file medinform_v4i1e3_app1.pdf]

Rule-of-thumb (RoT):

These algorithms are based on the difference between the current weight or NITTI measurement, and a previous measurement that occurred a predefined number of days in the past, as given by the following:

$$\text{Output index:} \qquad OI(t) = v(t) - v(t-d)$$

where *v* indicates the measurement value as a function of time, *t* indicates the time in days for which the output index (*OI*) is calculated and *d* is the parameter determining the time difference used in days.

This algorithm is analogous to guideline recommendations for weight monitoring proposed by the ESC [9]; where *v(t)* would be weight (in kg) today and *v(t-d)* would be the weight 3-days previously; with an alert being triggered if the output index exceeded 2 kg. In addition to the RoT weight algorithms proposed by the ESC and the HFSA, we have tested the RoT weight rules published in several other studies [21,23,26].

Moving average convergence divergence (MACD):

This algorithm, often used for stock price analysis, was previously tested by Zhang et al. [23] to predict decompensated HF, based on home-telemonitoring weight data. The algorithm calculates the difference between two exponentially weighted moving averages (EWMA) having different time spans. This approach is less sensitive to noise in the measurement and detects trends rather than short increases.

$$\text{Output index:} \qquad OI(t) = \alpha_s \sum_{x=0}^{\infty} (1-\alpha_s)^x v(t-x) - \alpha_l \sum_{x=0}^{\infty} (1-\alpha_l)^x v(t-x)$$

$$\alpha_s = \frac{2}{N_s + 1} \qquad \alpha_l = \frac{2}{N_l + 1}$$

where *v* indicates the measurement value (ie weight or NITTI), *t* indicates the time in days when the output index (*OI*) is calculated, the parameter N_s determines the length of the short time span average and the parameter N_l determines the long time span average. The first measurement was used to set values of *v* for all older measurements.

Cumulative sum control chart (CUSUM):

A statistical quality control technique used to monitor change was proposed by Adamson et al. [31] for the detection of decompensated HF using PAP. This method detects deviations in a specific direction from the recorded values by use of a cumulative sum of differences from a running average normalized for the noise as measured by the standard deviation. The directionality and the use of a slowly accumulated value makes this algorithm similar to the impedance algorithm used by Yu et al. [33] for ITI measurements.

$$\text{Output index:} \qquad OI(t) = \max\{0, \alpha + OI(t-1) - c\}$$

$$\alpha = \frac{v(t) - \text{mean}\begin{cases} v(x) \\ x: \{t, \ldots, t-d\} \end{cases}}{\text{std}\begin{cases} v(x) \\ x: \{t, \ldots, t-d\} \end{cases}}$$

where *v* indicates the measurement value as a function of time, *t* indicates the time in days for which the output index (*OI*) is calculated, the parameter *d* determines the length of the running mean and standard deviation and the parameter *c* determines the depreciation of the

accumulated sum. A minimum requirement of 5 values was required to establish the standard deviation.
